# The Behavioral Responses of Koi Carp (*Cyprinus carpio*) to Different Temperatures: Which Is Better, Infrared or Quadrupole Technology?

**DOI:** 10.3390/ani15070943

**Published:** 2025-03-25

**Authors:** Guoqing Zhong, Zongming Ren

**Affiliations:** College of Geography and Environment, Shandong Normal University, Jinan 250358, China; 2022021218@stu.sdnu.edu.cn

**Keywords:** behavior monitoring, water temperature, circadian rhythm, physiological ecological synthesis analysis, multi-modal monitoring

## Abstract

Based on the homemade Physiological and Ecological Comprehensive Analysis System for Aquatic Animals (PECA-BES01), this study compared the behavioral responses of koi carp (*Cyprinus carpio*) at three temperature gradients using two behavioral monitoring techniques as follows: infrared tracking and quadrupole impedance. The experiment employed comprehensive behavioral strength monitoring and infrared tracking (with tracked coordinates converted to swimming velocity data) to reflect behavioral changes. Within a certain temperature range, the behavioral strength and swimming velocity of carp increased with increasing temperature, which indicated heightened activity. The average behavioral strength and swimming velocity during light conditions (over three temperature gradients) were greater than during dark conditions. The circadian rhythm of carp becomes unstable at high temperatures, which shows abnormal periodicity with earlier occurrences of diurnal time points. Results from the system’s two behavioral monitoring methods were largely consistent and confirmed the reliability of PECA-BES01 in monitoring aquatic organism behavior. Simultaneously, each of the two technologies has its own characteristics. Quadrupole impedance can be used to monitor the behavioral response of fish to different water depths, whereas infrared tracking can be used to monitor the behavioral response of fish to different flow velocities. Therefore, both behavioral strength monitoring and infrared tracking monitoring are effective techniques for monitoring fish behavior and can be widely applied. This study provides scientific support for koi carp cultivation and other aquatic species aquaculture, while also aiming to deliver high-quality methodology for online monitoring of aquatic organisms.

## Simple Summary

This study highlights the potential for integrating multiple monitoring methods to improve the comprehensiveness and reliability of behavioral assessments in aquatic environments. Temperature is a pivotal environmental parameter for koi carp, and the study of its effects on the physiological processes of koi carp can provide a scientific basis for research on koi culture, as well as aquaculture of other fish and other aquatic organisms. This study can also validate the reliability of the laboratory’s self-developed online analysis system, which is expected to provide a high-quality means of online monitoring of aquatic organisms.

## 1. Introduction

Contemporary water quality surveillance systems predominantly rely on physicochemical analytical methods. The method is highly targeted and capable of accurately and scientifically detecting various harmful substances in water, such as heavy metals and organic pollutants [1]. However, these methods prove time-intensive for data collection and lack real-time alert capabilities, requiring costly laboratory procedures [2]. Current physicochemical analysis monitors individual parameters while neglecting critical biological factors, including pollutant bioavailability, synergistic interactions, and ecological impacts [3]. Furthermore, the approach faces constraints from low sampling frequency and substantial operational costs involving infrastructure maintenance, specialized personnel, and per-analysis expenditures [4]. In contrast, online biological monitoring can effectively address such issues. Biological responses reflect the strength of environmental pressure and can serve as early warning indicators when organisms begin to experience adverse effects [5]. Furthermore, online biological monitoring is also highly cost-effective, has the ability to continuously monitor stress responses of organisms under environmental insults, and has many other advantages [6,7].

Aquatic environmental alterations directly induce behavioral modifications in fish populations [8]. Organisms counteract environmental stressors through cellular-level biochemical responses necessitating elevated metabolic energy production [9,10]. Elevated metabolic activity inevitably augments reactive oxygen species (ROS) generation oxygen-derived intermediates, including superoxide (O^2−^), hydrogen peroxide (H_2_O_2_), and hydroxyl radicals (HO^•^) [11]. This overproduction triggers oxidative stress when ROS accumulation surpasses endogenous antioxidant capacity [12], subsequently damaging lipids, DNA [11], and altering organismal behavior [13]. Liu et al., demonstrated that formaldehyde exposure triggers characteristic neurobehavioral alterations in *Danio rerio*, manifesting as erratic locomotion (erratic turning/thigmotaxis) and restricted movement patterns [14]; additionally, Brunelle et al. demonstrated wastewater effluent containing organic contaminants (acetaminophen, bupropion, caffeine) induced hyperactivity in *Danio rerio* larvae during colder seasons (autumn–winter) [15]. Fish have been historically employed as real-time biomonitors due to their ease of aquaculture, availability, suitable specimen size, and lifespan [4]. Furthermore, their highly developed central nervous systems exhibit sensitivity to diverse industrial pollutants (e.g., heavy metals, herbicides, neurotoxins), and their behavioral responses holistically reflect the combined toxicity of multiple contaminants in real time [16,17]. Therefore, monitoring fish behavior is a reliable way to understand the current water environment. In contemporary research, the quantitative analysis of fish behavior has emerged as a foundational element in various investigative domains, including biological water quality monitoring [1,2,8]. Additionally, the quantitative assessment of piscine behavioral patterns plays a crucial role in the design and operation of aquaculture systems [18]. Overall, this analytical approach has demonstrated significant utility across multiple disciplines within the aquatic sciences.

Presently, online monitoring techniques based on behavioral biology primarily employ video tracking systems and quadrupole impedance monitoring systems [19]. Alternative methodologies, such as bivalve mollusk, luminescent bacteria, and opercular monitoring, among others, are less frequently utilized [20,21]. Moreover, the majority of the current applications rely on a single monitoring technique with the simultaneous implementation of dual monitoring technologies within a unified system being virtually nonexistent in the literature. This observation underscores the potential for integrating multiple monitoring approaches to improve the comprehensiveness and reliability of behavioral assessments in aquatic environments.

With the development of water conservancy and increasing demands for flood control, irrigation, and power generation, the construction of hydraulic structures, such as dams, weirs, and sluices, in rivers has rapidly increased [22]. While dams result in significant economic benefits, their ecological impacts cannot be ignored. These include changes in river flow patterns, sediment dynamics [23], upstream and downstream geomorphology [24], and obstructions to migration routes [25,26,27]. Among these factors, the blockage of migration routes is a critical impact of dams on fish and aquatic mammals [28]. Migration barriers disrupt critical spawning cycles, reducing fish and aquatic mammal biodiversity through altered flow regimes, particularly in dam-proximal river zones. For example, the ten dams that are currently under construction in the upper Yangtze River will block the migration routes of 35 species of migratory fish, such as the Chinese sturgeon (*Acipenser sinensis*) [29]. This will increase the extinction risk for species such as the largemouth bronze gudgeon (*Coreius guichenoti*) and reduce the likelihood of capturing species such as the Yangtze sturgeon (*Acipenser dabryanus*) and the white sturgeon (*Psephurus gladius*) [30]. Therefore, restoring fish migration pathways is crucial for protecting river biodiversity. Fishways are effective tools for reestablishing migration routes and connecting fragmented habitats [31]. However, fish that navigate these passages are influenced by various factors, including flow velocity, turbulence [32,33], and water temperature [34].

Temperature, which is a critical environmental parameter, significantly influences the metabolic processes of aquatic organisms [35]. Temperature fluctuations have the potential to exert profound effects on a diverse array of biological activities, encompassing lifestyle patterns [36], locomotor capabilities [37], swimming behaviors [38], feeding and reproductive processes [39], predator–prey dynamics [40], and, most critically, longevity [41]. For example, fluctuations in temperature and dissolved oxygen (DO) concentrations elicit diverse effects on the oxygen supply, metabolite flux, and intracellular adenosine triphosphate (ATP) and phosphocreatine (PCr) reserves, as well as endogenous fuel stores within white muscle tissue [42]. Water temperature critically regulates upstream migration in various fish species [43]. Migration onset correlates with species-specific thermal optima, though thresholds vary considerably within and between taxa [44]. Suboptimal temperatures below these thresholds impair migration initiation, reducing efficiency in fish. Thermal windows governing peak migratory performance highlight temperature’s dual role in both initiating and optimizing fish movement [45]. Furthermore, across all vertebrate taxa, the molecular mechanisms governing circadian rhythmicity involve transcriptional–translational feedback loops between circadian genes and their corresponding proteins [46]. These intricate regulatory networks are modulated by various environmental cues, including temperature cycles [47,48], light–dark periodicity [49], and feeding schedules [50].

Koi carp (*Cyprinus carpio*) constitute a high-value ornamental species supporting a transnational industry across major production zones (China, Southeast Asia, Europe, and North America). Market valuations indicate a global koi trade valued at USD 2.3 billion (2023) [51]. Koi carp, which are poikilothermic organisms, lack the ability to regulate their body temperature autonomously. Furthermore, the efficiency of metabolic processes within their bodies is intrinsically dependent on their body temperature. The routine metabolic rate (RMR) of carp (which serves as an index of an organism’s oxygen demand and resting energy requirements) significantly increases with rising rearing temperatures, and temperature also induces gill remodeling in carp to enhance oxygen uptake capacity [52]. Additionally, vitamin E, and vitamin C [53], as well as wind and water level fluctuations, all influence carp growth and population abundance [54]. Notably, under the context of global warming, fish exhibit accelerated growth rates accompanied by reduced body size and an earlier age of maturation [55,56]. Temperature is one of the most critical environmental variables influencing the interactions between poikilothermic organisms and pathogens [57,58]. Consequently, the selection of habitats with optimal thermal conditions for fish is paramount. Such environmental optimization serves two purposes in koi carp: maintaining normal metabolic functions while simultaneously maximizing immunological defenses against diseases [59]. Furthermore, temperature fluctuations significantly influence various physiological parameters in koi carp, including plasma carotenoid levels and related metabolites, which consequently affect their skin coloration [60]. The vivid body coloration and exquisite color patterns of koi carp represent their most significant economic traits [51], serving as critical indicators for assessing fish quality and determining market value [61]. Given these multifaceted effects, the temperature has emerged as a critical environmental parameter for koi carp with far-reaching implications for their physiological processes and esthetic qualities.

In response to the aforementioned research findings, we developed a Physiological and Ecological Comprehensive Analytical System for Aquatic Animals (PECA-BES01). The primary objectives of our study are twofold: first, to evaluate the efficacy of our custom-designed system, and second, to quantify the real-time responses of koi carp to various environmental fluctuations through a comparative analysis of two distinct behavioral monitoring methodologies. In this investigation, koi carp specimens were subjected to three discrete temperature gradients. This study employs quadrupole impedance technology in conjunction with infrared tracking techniques to conduct online monitoring of the behavioral responses exhibited by koi carp. These responses were subsequently subjected to quantitative analysis.

## 2. Materials and Methods

Ethical approval for this research was granted by the Animal Experiment Ethics Review Committee of Shandong Normal University (Approval No. AEECSDNU2024141, Date: 2024).

### 2.1. Experimental Apparatus

**Figure 1:**
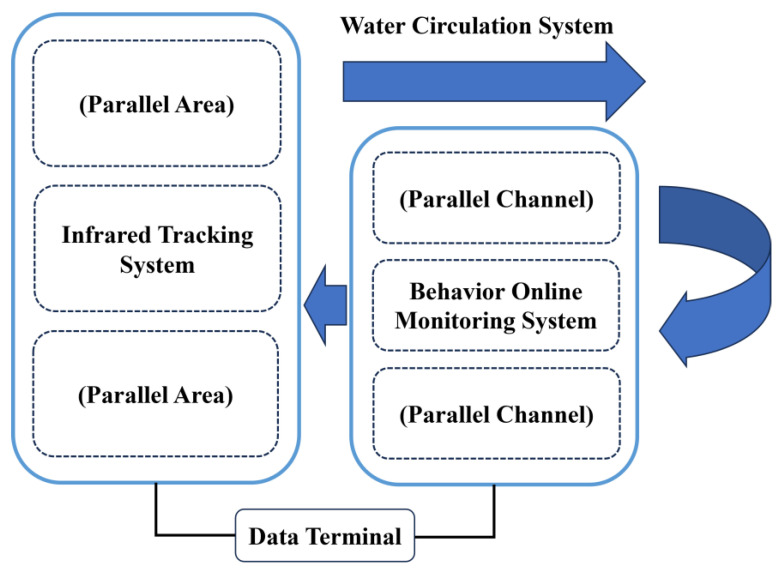
Schematic diagram of the Physiological and Ecological Comprehensive Analysis System for Aquatic Animals.

The experimental apparatus employed in this study is the Physiological and Ecological Comprehensive Analysis System for Aquatic Animals (PECA-BES01, Jinan, China). Current commercial fish behavior monitoring systems predominantly rely on computer vision technologies. However, these systems encounter recognition errors and tracking failures when confronted with obstructions causing target occlusion, ambient light intensity variations, overlapping monitored targets, or individual motion blur [62]. In contrast, the PECA-BES01 system employs quadrupole impedance technology that circumvents such limitations, while uniquely integrating infrared tracing monitoring technology compared to other quadrupole impedance systems available. Through the synergistic application of these dual technological approaches, PECA-BES01 achieves enhanced parameter diversity acquisition, enabling comprehensive behavioral analysis of aquatic organisms. Furthermore, its modular architecture permits component customization according to the morphological characteristics of different species. This innovative system was independently developed and manufactured by the Institute of Environment and Ecology at Shandong Normal University. The PECA-BES01 comprises three integral components (Figure 1): an infrared tracking system, an online behavioral monitoring system, and a water circulation system. The system has been designed to allow the online monitoring of fish with a range of body sizes.

#### 2.1.1. Infrared Tracking System

A miniature online electrocardiogram acquisition device is placed on the fish to collect signals, which are then transmitted in infrared form. The signal-receiving end consists of 3 × 4 receiving router boards, and each is equipped with 5 × 5 miniature signal-receiving devices capable of accepting electrocardiogram data and infrared receiver module information. These signals are converted into analog signals in real time and transmitted to the Pclab-530C (Beijing Micro Signal Star, Beijing, China) biomedical signal acquisition and processing device, which subsequently transmits the acquired data to the main interface of the terminal. The terminal records the fish’s coordinates (X, Y) every 10 s and determines the central position of the fish’s body through a centroid algorithm. The swimming velocity of the fish is calculated using the fish coordinates and time data.

#### 2.1.2. Behavior Online Monitoring System

Based on quadrupole impedance technology, the system comprises three biosensors and a monitoring acquisition control terminal. Commonly used fish behavior monitoring instrument technologies mainly include computer vision monitoring technology and quadrupole impedance monitoring technology. Compared with computer vision technology, quadrupole impedance technology avoids issues such as light interference, high costs, and complex data processing [63]. Liu et al., utilized this technology to discover that cadmium stress inhibits zebrafish behavior, with the behavioral responses showing distinct 24 h rhythmicity [64]. Similarly, He et al. applied the same technology to demonstrate suppressed behavioral strength in zebrafish under ammonia–nitrogen stress [65]. These studies confirm that this technology features real-time monitoring capabilities and delivers precise, effective results. The inner wall of each biosensor contains two pairs of platinum electrodes as follows: one pair serves as the emitting electrode and generates a low-voltage, high-frequency alternating current signal (voltage 0.5–5 V, frequency 100 Hz) to create an electric field; the other pair functions as receiving electrodes capable of detecting disturbances produced by fish swimming in the electric field [66]. The monitoring acquisition control terminal processes the collected digital signals through Fast Fourier Transform (FFT) into visible signals [67] and generates corresponding curves on the computer display interface. The behavioral strength range of the fish is [0–1], where 0 indicates relative immobility or loss of vitality, and 1 represents the maximum activity level of the fish [68].

#### 2.1.3. Water Circulation System

The system is equipped with a 12 V, 600 W brushless motor propeller (Haibo Power Industry, Ningbo, China), which can control the water flow velocity according to experimental requirements and maintain water circulation throughout the entire system. In this experiment, the water flow was controlled at 0.06 m/s and provided sufficient dissolved oxygen for the experimental channel. The circulation system also includes a set of automatically controlled sliding doors, which can regulate the presence or absence of water flow and aerobic or anaerobic environments in the experimental channel. For the behavioral strength monitoring experiment, a 45 min no-flow condition was set, which was followed by a 15 min flow condition to oxygenate the experimental channel.

#### 2.1.4. Experimental Subject: Koi Carp

The koi carp (20–30 cm) selected for this experiment were sourced from the laboratory of the Institute of Environment and Ecology at Shandong Normal University. The laboratory is equipped with a comprehensive scientific breeding system, which includes a water circulation and filtration system and an oxygenation system. The water temperature was maintained at 22 °C ± 0.5 °C. Fish were fed twice a day (at 7:00 AM and 4:00 PM), and the photoperiod was set to 16 h of light and 8 h of darkness (16L:8D), controlled by an automated device.

### 2.2. Experimental Design

The experiment consisted of two parts as follows: monitoring the behavioral strength and tracking the behavior of koi carp. Each part was divided into three groups, which were at 18 °C, 22 °C, and 26 °C. Each experimental group included three replicates. The laboratory fish were consistently maintained in a 22 °C water environment, which is the optimal growth temperature for koi carp. Based on studies by Xu, Yang, and others [69,70], temperature differences of 4–5 °C significantly affect koi respiration rates and metabolic differences. To account for varying experimental outcomes under different temperature gradients, a higher temperature (26 °C) and a lower temperature (18 °C) were set.

The experimental procedure was as follows: ① Experimental water (tap water) was added to the tank, which was then thoroughly aerated for more than 3 days to eliminate residual chlorine and ensure sufficient dissolved oxygen. The experimental tank was connected to a filtration box, which utilized materials such as fiber cotton and ceramic rings for filtration. The water hardness (CaCO_3_) was maintained at 250 ± 25 mg/L (using an online water hardness analyzer, ERun-SZ-3085, Xi’an, China) with a pH of 7.8 ± 0.2 (using a Five Easy pH meter, JJG 119, Shanghai, China). The total volume of experimental water was approximately 3500 L. ② Selection of experimental subjects was as follows: four healthy and active koi carp, measuring 20–30 cm in length, were randomly selected. Three of these fish were placed individually into three separate channels. The fourth fish was anesthetized in advance with tricaine methane sulfonate (MS-222). Anesthesia was administered by adding 1.5 mL of MS-222 to 4 L of water [5,71]. Once the fish were successfully anesthetized, a miniature online electrocardiogram acquisition device was attached to its dorsal area. The anesthetized fish was then placed in the PECA-BES01 electrocardiogram zone and allowed 30 min to recover from the anesthesia and return to a normal state. The koi carp were not fed for one day prior to the experiment. ③ On the day preceding the experiment, heating rods and automatic control equipment were utilized to regulate the water temperature within the intended testing range. Prior to the experiment, the PECA-BES01 was activated, and the program was initiated. After 10–20 min, a calibration was performed. The automatic control device for the sliding doors of the PECA-BES01 was activated with time intervals set as follows: 15 min for oxygenation with water flow and 45 min without oxygenation and water flow. The PECA-BES01 collected and recorded experimental data for 48 h and commenced at 10:00 a.m. on the first day and concluded at 10:00 a.m. on the third day. The utilized light source was a manually controlled daylight lamp with a strength range of 3000 to 4500 lx. The photoperiod was set from 6:00 to 20:00 in the daytime, with a night period from 20:00 to 6:00 the following day, which resulted in a light–dark cycle ratio of 14 h:10 h. Fish were not fed during the experimental period. The experimental area was located away from populated zones, and access was restricted during the experiment to prevent unauthorized personnel from approaching. Additionally, the system was surrounded by soundproofing foam to effectively minimize ambient noise interference. All instruments were surrounded or lined with sponge padding to minimize mechanical vibrations generated during operation.

### 2.3. Analytical Methods

This study utilized Microsoft Excel 2021 to process data obtained from PECA-BES01 and performed calculations for swimming velocity, mean values, and variance analysis. Data collected from infrared tracking were converted from coordinates to swimming velocity using the following formula:

(1) $$v=\frac{\sqrt{{\left(x_{1}-x_{2}\right)}^{2}+{\left(y_{1}-y_{2}\right)}^{2}}}{t}\times 5$$

Among these, v represents the swimming velocity of fish and is measured in cm/s; x_1_ and y_1_ denote the coordinates of the fish’s center position in the first second; x_2_ and y_2_ represent the coordinates of the fish’s center position after 10 s; t is the data collection interval, which is 10 s; and 5 is a constant, which indicates the unit distance between each coordinate point is 5 cm.

Behavioral metrics were recorded at 60 s intervals through automated monitoring systems. Triplicate experiments conducted across three thermal gradients (18 °C, 22 °C, 26 °C) generated parallel datasets.

Statistical analysis using Microsoft Excel calculated means and standard deviations, with final data expressed as mean ± SD for both behavioral intensity and swimming velocity across temperature conditions. Behavioral strength metrics were standardized as: ABSV (average behavioral strength value), ABSVL (light periods average), and ABSVD (dark periods average) [64,65]. Swimming velocity metrics were standardized as: AV (average velocity), AVL (light periods average), and AVD (dark periods average) [72,73]. Behavioral strength and swimming velocity differentials between photoperiods were calculated as:

D (ABSVL − ABSVD) = (ABSVL − ABSVD)/ABSVD × 100%

D (AVL − AVD) = (AVL − AVD)/AVD × 100%

The mean data were processed and plotted using Origin 2024.

Statistical analyses were conducted using IBM SPSS 25.0, incorporating Shapiro–Wilk normality tests, independent *t*-tests, and one-way and two-way ANOVA. Histograms provided visual confirmation of normal distribution across datasets. Behavioral metrics (ABSV) and locomotor performance (AV) comparisons between photoperiods revealed significant differences at *p* < 0.05 or *p* < 0.001.

MATLAB R2024a was employed to perform autocorrelation analysis and Self-Organizing Map (SOM) analysis on the processed data. These analyses were used to visually examine circadian rhythm changes in koi carp and variations in behavioral strength and swimming velocity under different temperature gradients.

## 3. Results

### 3.1. Behavioral Responses of Koi Carp

**Figure 2:**
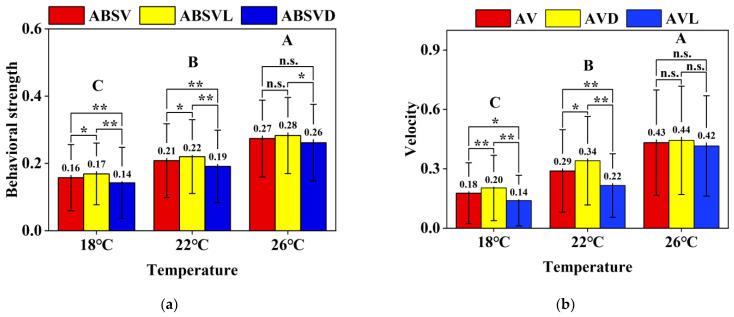
Differences in the average behavioral strength of koi carp under three temperature gradients across photoperiods (**a**) (ABSV-Average Behavioral Strength Values, ABSVL-Average Behavioral Strength Values in Light periods, ABSVD-Average Behavioral Strength Values in Dark periods) and differences in the average velocity of the photoperiods of koi carp under three temperature gradients (**b**) (AV-Average Velocity, AVL-Average Velocity in Light periods, AVD-Average Velocity in Dark periods). N = 27, data were present as means ± SD. * Indicates significant differences in the light and dark periods. * means *p* < 0.05, ** means *p* < 0.001, n.s. means *p* > 0.05. Letters A, B, and C represent differences among temperatures, *p* < 0.001.

Figure 2a,b illustrate the effects of temperature and photoperiod (light/dark periods) on the average behavioral strength and swimming velocity of koi carp over 48 h trials, analyzed via two-way ANOVA with temperature and photoperiod as main effects.

(a) A significant main effect of temperature was observed (*p* < 0.001), with behavioral strength increasing progressively across temperature gradients: 0.16 ± 0.10 (18 °C) < 0.21 ± 0.11 (22 °C) < 0.27 ± 0.11 (26 °C). Photoperiod exerted a significant main effect at 18 °C (*p* < 0.001) and 22 °C (*p* < 0.001), following the hierarchy ABSVL > ABSV > ABSVD (21.4% and 15.8% differences, respectively). However, this photoperiod effect diminished at 26 °C (7.7% difference, *p* < 0.05), indicating a significant temperature × photoperiod interaction (*p* < 0.05).
(b) Similar thermal dependence was evident in swimming velocity (*p* < 0.001): 0.18 ± 0.15 cm/s (18 °C) < 0.29 ± 0.21 cm/s (22 °C) < 0.43 ± 0.27 cm/s (26 °C). Photoperiod significantly influenced velocities at 18 °C (42.9% difference, *p* < 0.001) and 22 °C (54.5%, *p* < 0.001), adhering to the AVL > AV > AVD hierarchy. Notably, the temperature × photoperiod interaction was significant (*p* < 0.05), as photoperiod effects vanished at 26 °C (4.8% difference, *p* > 0.05), with no diurnal activity variation observed.

**Figure 3:**
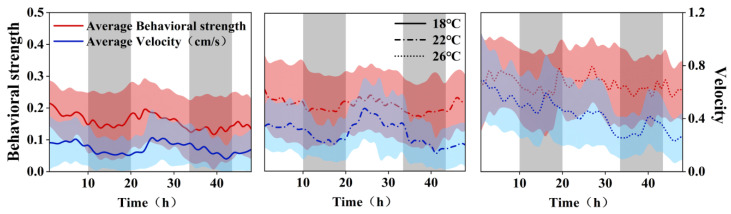
The behavioral strength and velocity curves of koi carp under three temperature gradients.

Koi carp demonstrated temperature-dependent circadian rhythm modulation in their behavioral patterns (Figure 3). At 18 °C and 22 °C, individuals maintained stable 24 h activity cycles characterized by light periods behavioral peaks and dark periods troughs, with significant light/dark differentiation (18 °C: 21.4%, 22 °C: 15.8%, *p* < 0.001). However, 26 °C exposure induced circadian rhythm fluctuations marked by dual activity peaks during dark periods and altered photoperiod entrainment—light periods initiation advanced approximately 4 h earlier than theoretical values while maintaining total duration. Swimming velocity paralleled these thermal effects, showing light period elevations over dark periods at 18–22 °C (*p* < 0.001), but losing circadian differentiation at 26 °C (4.76% variation, *p* > 0.05). This thermal disruption preserved baseline 24 h periodicity but compromised phase alignment and amplitude control, suggesting partial circadian desynchronization under elevated temperatures.

The experimental results across three temperature gradients reveal that both the behavioral strength and swimming velocity of koi carp exhibit a positive correlation with temperature elevation. At 18 °C and 22 °C, significant differences in these parameters are observed between light and dark phases, with a discernible periodicity. However, at 26 °C, the diurnal variations in koi carp behavior diminish, and the circadian rhythm is disrupted.

### 3.2. Autocorrelation Analysis

**Figure 4:**
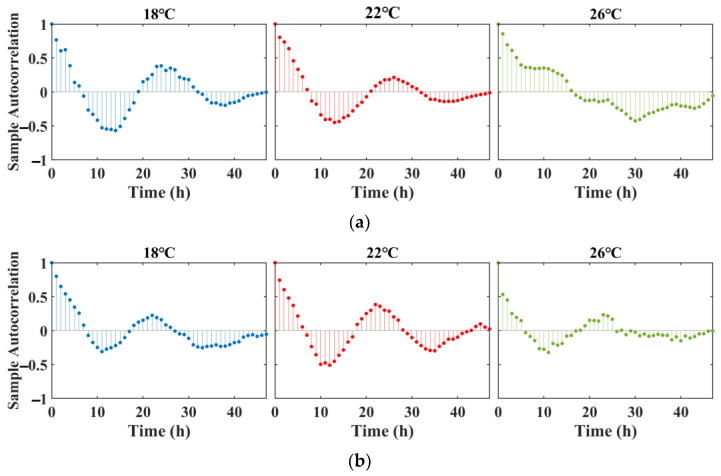
Autocorrelation function applied to the BS of koi carp under three temperature gradients (**a**) and the velocity of koi carp under three temperature gradients (**b**).

Figure 4a,b depict the autocorrelation analysis of koi carp’s behavioral strength and swimming velocity across temperatures of 18 °C, 22 °C, and 26 °C. Autocorrelation analysis can detect whether the data exhibit periodic fluctuations. At 18 °C and 22 °C, both parameters show periodic positive and negative correlations, near symmetry, and smooth, stable fluctuations. At 26 °C, however, the circadian rhythm anomalies become evident: the behavioral strength’s autocorrelation loses its periodicity, the positive and negative correlations become asymmetrical, and the trends fluctuate more dramatically. The slightly higher negative correlation in the first cycle and positive correlation in the second cycle at 22 °C compared to 18 °C suggest that the koi carp’s behavioral patterns are more pronounced and stable at 22 °C. This may be due to the fact that 22 °C is closer to the optimal temperature for koi carp [70], enhancing their activity and resulting in more regular behavioral cycles.

### 3.3. SOM Analysis

**Figure 5:**
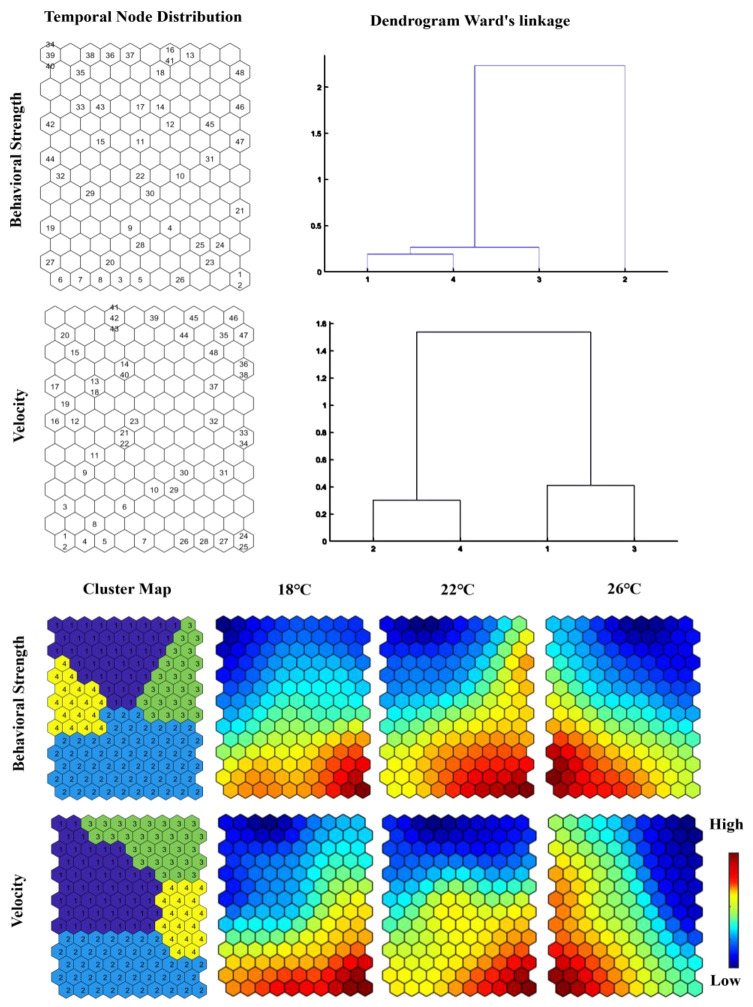
SOM patterns of koi carp behavioral strength and velocity.

The circadian behavioral patterns of koi carp under varying temperatures were analyzed using SOM clustering of hourly averaged behavioral strength and swimming velocity, with comparative results detailed in Figure 5.

Cluster 2, associated with light periods, displayed heightened activity (red zones), whereas Clusters 1, 3, and 4 (dark periods) corresponded to reduced activity (blue zones). Both 18 °C and 22 °C conditions showed overlapping high-activity patterns, suggesting conserved circadian rhythms at these temperatures. Notably, the 22 °C condition exhibited slightly expanded red zones compared to 18 °C, implying temperature-dependent amplification of behavioral strength. At 26 °C, while Cluster 2 retained its association with light periods, the temporal distribution of high-activity phases shifted forward, suggesting their biological timing system has become disconnected from day–night signals.

Clusters 2 and 4 (light periods) corresponded to elevated velocity (red zones), contrasting with Clusters 1 and 3 (dark periods, blue zones). Similarly to behavioral strength patterns, 18 °C and 22 °C conditions maintained a steady circadian rhythm. However, at 26 °C, the system exhibited dual disruptions: a phase advance in light-period activity and anomalous activation during dark periods (Cluster 1), demonstrating rhythmic fragmentation.

Lower temperatures (18–22 °C) modulated behavioral amplitude without compromising circadian stability, whereas 26 °C induced systemic circadian disruption. Key alterations at 26 °C included phase-advanced activity onsets, intrusion of elevated activity into dark periods, and desynchronization of rhythmic components. These combined effects—temporal misalignment, arrhythmic activation, and rhythm fragmentation—collectively suggest a breakdown in circadian architecture under elevated thermal conditions.

### 3.4. Comparative Analysis

Quadrupole impedance (behavioral strength) and infrared tracking (swimming velocity) monitoring demonstrated concordant circadian patterns across thermal gradients. Both methods captured light-phase increases and dark-phase decreases in activity metrics at 18 °C and 22 °C, with circadian rhythms becoming phase-advanced (~4 h) and attenuated at 26 °C. While trends aligned temporally, quantitative stability differed significantly: behavioral strength measurements showed lower variability (0.16 ± 0.10 to 0.27 ± 0.11) compared to velocity data (0.18 ± 0.15 to 0.43 ± 0.27 cm/s), indicating greater methodological consistency in impedance-based monitoring versus infrared tracking.

**Figure 6:**
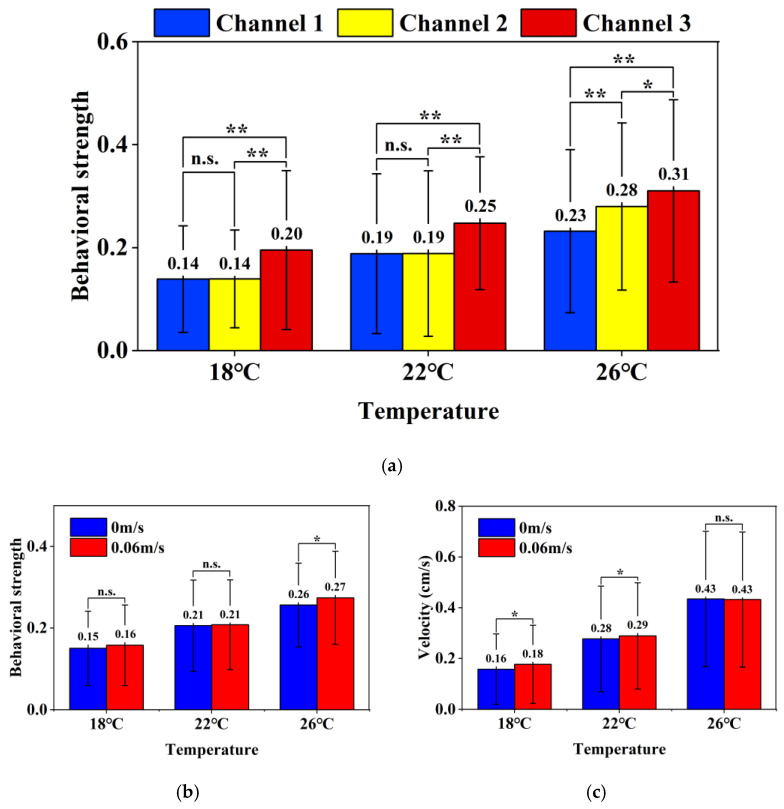
Comparison of the behavioral strength of koi carp at different water depths (**a**), comparison of the average behavioral strength of koi carp at different flow velocities (**b**), and comparison of the average velocity of koi carp at different flow velocities (**c**). N = 27, data were present as means ± SD. * means *p* < 0.05, ** means *p* < 0.001, n.s. means *p* > 0.05.

Figure 6a demonstrates depth-dependent behavioral intensity variations in carp across thermal gradients. At all temperatures (18 °C/22 °C/26 °C), surface-proximal specimens (200 mm depth) exhibited greater behavioral strength than deeper counterparts (450/700 mm) (*p* < 0.001). Significant depth effects emerged only at 26 °C for mid-depth comparisons (450 vs. 700 mm, *p* < 0.001), while 18 °C/22 °C showed no statistical distinction between these deeper strata. The experimental design utilized fixed vertical gradients (700/450/200 mm) achieved through hydraulic calibration rather than structural modification. In addition, Figure 6a reveals depth-stratified thermal responsiveness in koi carp behavior. At all depths (200/450/700 mm), behavioral strength exhibited positive thermal correlations (18–22–26 °C), aligning with established temperature-dependent activity patterns. Crucially, shallower depths (200 mm) amplified behavioral responses compared to deeper zones (*p* < 0.001), demonstrating water column position significantly modulates thermal-responsive behavioral modulation in carp. Figure 6b demonstrates minimal hydrodynamic influence on carp behavioral strength. Comparative analyses of static (0 m/s) versus flow conditions (0.06 m/s) across thermal gradients revealed non-significant differences (18 °C/22 °C, *p* > 0.05), except at 26 °C, where flow exposure marginally increased behavioral strength (*p* < 0.05). This suggests quadrupole impedance monitoring maintains stability under low-flow environments (≤0.06 m/s). Figure 6c delineates temperature-dependent rheotactic responses. At 18 °C/22 °C, flow conditions significantly elevated swimming velocities (*p* < 0.05), whereas 26 °C specimens showed flow-compensated locomotion (*p* > 0.05). These results indicate that thermal thresholds modulate hydrodynamic sensitivity in koi carp, with elevated temperatures potentially overriding flow-induced locomotor adaptations.

## 4. Discussion

Behavior is an effective indicator that helps us understand the physiological and ecological status of organisms in their environment [8]. Therefore, we can utilize the behavior of organisms to assess the effects of their exposure to environmental stressors [74]. Continuous monitoring of Cyprinus carpio behavior quantified temperature-dependent activity patterns across thermal gradients (18 °C/22 °C/26 °C). Both metrics exhibited parallel thermal responses, with behavioral strength (ABSV) increasing 68.8% (18 → 26 °C) and swimming velocity (AV) surging 138.9% (18 → 26 °C). This result may be related to the fact that koi carp are poikilothermic. An increase in environmental temperature leads to an increase in fish body temperature, which subsequently affects the efficiency of metabolism. Changes in koi carp metabolism can be directly reflected by their oxygen consumption rate [75]. Therefore, this study indicates that within a certain range and as the temperature increases, the oxygen consumption rate of koi carp increases, which is consistent with the findings of Yang et al. [69].

Koi carp demonstrate endogenous circadian entrainment synchronized with photoperiodicity, as evidenced by congruent behavioral (ABSV) and locomotor (AV) monitoring datasets. However, at 26 °C, koi carp behavioral responses are disrupted and manifest as abnormal circadian rhythms with advanced timing of activity. This phenomenon may be related to the oxygen consumption rate of koi carp. The stable range for the oxygen consumption rate of koi carp is 20–25 °C [69]. At 26 °C (beyond thermal optima), koi carp exhibit paradoxical declines in oxygen consumption, indicating metabolic suppression that impairs behavioral regulation through compromised aerobic capacity. Moreover, at 18 °C, the smaller light–dark cycle difference and lower ABSV and AV indicate fewer active koi carp behavior. Thus, 22 °C seems more suitable for their survival. This result is similar to the findings of Chen and others [70]. For commercial koi farming, maintaining water temperature at 22 °C enhances growth and development. This is in line with the results of practical farming, where the optimal water temperature for koi carp is about 23 ± 1 °C [76,77]. In addition, the behavior of fish such as Giant danios (*Devario aequipinnatus*) and European sturgeon (*Acipenser sturio*) is also affected by temperature, which includes, but is not limited to, changes in cohesion, alterations in activity, and variations in swimming speed [78,79].

While the two koi carp monitoring methods yield closely aligned results, notable analytical variations persist. Quadrupole impedance-based behavioral strength measurements show lower rate changes compared to infrared tracking data. At 26 °C, koi carp exhibited 28.57% higher mean behavioral strength than at 22 °C and 68.75% greater than at 18 °C, with inter-temperature variations being less pronounced than infrared-derived measurements. Corresponding velocity measurements revealed 48.28% and 138.89% increases at 26 °C relative to 22 °C and 18 °C, respectively. These findings do not suggest methodological superiority. The impedance technique quantifies behavioral strength through electric field perturbations, employing signal filtration [66] to produce interference-resistant datasets with stable analytical progression. Conversely, infrared tracking relies on centroid coordinate detection, where minor positional deviations directly affect measurements. This approach demonstrates heightened sensitivity to movement dynamics, yielding more pronounced analytical fluctuations that may correlate with environmental noise artifacts. In addition, two techniques have their own characteristics, such as quadrupole impedance technology, which is designed with three channels in parallel from top to bottom so that the behavioral response of the fish can be monitored at different water depths, and results show that the behavior of koi carp is inhibited by water depth within a certain range of water depths. This ecological preference may stem from cyprinids’ functional dependence on shallow-water habitats. Carp naturally prefer shallow waters for spawning and feeding, as shown in Zhang’s river studies [80]. They dive deeper only to escape predators. Similar depth effects exist in other fish. The likelihood of *Maccullochella peelii* fatiguing more easily and exhibiting longer swimming endurance decreases significantly with increasing water depth [81]. Moreover, water depth may indirectly affect fish behavior by affecting enzyme activities in fish [82]. The bathymetric impacts on ichthyofauna provide critical insights for developing strategic management frameworks in fish population conservation and aquatic ecosystem stewardship.

While quadrupole impedance technology effectively quantifies water depth effects on fish behavior, it exhibits reduced sensitivity in real-time flow velocity monitoring compared to infrared tracking. The latter demonstrates superior efficacy in detecting flow-induced behavioral responses, reliably identifying measurable alterations in carp activity even at ultralow velocities (0.06 m/s). However, infrared signal propagation constraints preclude depth-related behavioral analysis, as signal reception requires proximity ≤ 500 mm between fish and receivers. Experimental constraints required maintaining this critical distance to ensure data acquisition fidelity. This limitation may be mitigated through hardware reconfiguration (e.g., arrayed receivers) or signal amplification protocols, enabling comprehensive depth-dependent behavioral monitoring.

Temperature affects fish behavior in diverse ways. These include lifestyle [36], locomotor ability [37], swimming patterns [38], feeding, reproduction [39], predator–prey dynamics [40], and most importantly, lifespan [41]. The fundamental reason why temperature can influence various fish behaviors may essentially be related to gene expression. As poikilothermic animals, fish can adapt to changes in environmental temperature. Although the molecular mechanisms behind this adaptation are not yet fully understood, studies have shown that fish exhibit differential gene expression when adapting to their environment. For example, Ju et al. analyzed 660 genes in catfish (*Ictalurus punctatus*) and reported that 61 genes were differentially expressed at 12 °C and 24 °C [83]. These include transcription factors and gene products involved in signal transduction pathways (such as zinc finger proteins and calmodulin kinase inhibitors), as well as genes involved in lipid metabolism (such as *TB2* and acyl-CoA binding proteins), among others. Zhou et al. discovered that tilapia resist cold by altering the expression of genes related to metabolism and immunity [84].

Additionally, the temperature can indirectly affect fish swimming ability by influencing their physiological metabolic processes, which in turn affects passage efficiency. For example, Penghan et al. found that low temperatures suppress crucian carp’s (*Carassius auratus*) swimming ability, reducing their speed and efficiency. Conversely, at higher temperatures, their swimming ability improves with increased speed and efficiency [85]. Guo Ziqi et al., demonstrated that *Schizothorax prenanti* exhibited >50% fishway passage efficiency within 13–21 °C, although regression analysis revealed an inverse thermal correlation [34]. Thermoregulatory demands, concurrent with hydraulic parameters (depth/flow velocity), constitute critical determinants in fish upstream migratory behavior necessitating systematic integration into fishway engineering design, as empirically validated by Baumann et al. [86].

In the context of global climate warming, water temperatures in natural environments are also changing. Based on the behavioral responses of fish to water temperature changes and their climate vulnerability [87], we must consider the impact of climate change on fish populations. Research has shown that in response to water temperature changes caused by climate change, fish often migrate to higher latitudes, leading to changes in their distribution ranges [88,89,90], as well as a reduction in average body size [91]. The rising temperatures exacerbate these challenges by increasing the anabolic oxygen demand of organisms while reducing oxygen solubility in water. Since smaller individuals face a lower risk of hypoxia, this ultimately drives a reduction in the average body size of fish populations over extended time scales [92]. Some aquatic organisms struggle to adapt to climate change, resulting in their extinction [93]. Therefore, further research is needed on how temperature specifically affects fish behavior.

## 5. Conclusions

Within a certain temperature range, both the behavioral strength and swimming velocity of koi carp increase as temperature increases, which indicates more active behavior. Koi carp exhibit distinct circadian rhythms with the average behavioral strength and average swimming velocity during light periods being greater than those during dark periods across all three temperature gradients. At 18 °C and 22 °C, circadian rhythms are stable over a 24 h period with rhythm at 22 °C being particularly pronounced. An SOM analysis confirmed that the behavioral strength and swimming velocity data of koi carp conform to temporal distribution patterns. At 26 °C, the circadian rhythm of koi carp becomes disrupted and results in abnormal periodicity and advanced timing activity. Results from behavioral strength monitoring and infrared tracking monitoring are generally consistent, although some differences exist, which may be due to the different monitoring principles used. Data obtained through quadrupole impedance technology are more stable, whereas those obtained via infrared tracking are more sensitive. Quadrupole impedance techniques can monitor the behavioral response of fish to different water depths, whereas infrared tracer techniques can monitor the behavioral response of fish to different flow velocities. Both behavioral strength and infrared tracking monitoring are effective techniques for monitoring fish behavior and can be widely applied.

The PECA-BES01 system demonstrates operational stability, maintenance simplicity, and multi-parametric monitoring accuracy for aquatic organisms. Its modular architecture allows customization across taxonomic groups, extending beyond cyprinids to include aquaculturally significant species (*Ctenopharyngodon idella*, *Carassius auratus*, *Pelteobagrus fulvidraco*), as well as decapod crustaceans and semi-aquatic reptiles. Future development roadmaps include embedded AI modules, a carbon-fiber-reinforced lightweight architecture (target mass reduction: 42%), and the integration of real-time metabolic profiling systems via dissolved oxygen/ammonia-nitrogen microsensors (resolution: 0.01 mg/L). Validation protocols for pelagic species (>1.5 m TL) and high-flow environments (≥2.5 m/s) are prioritized to establish a unified biomonitoring platform.

## Data Availability

The original contributions presented in the study are included in the article, further inquiries can be directed to the corresponding authors.

## Abbreviations

The following abbreviations are used in this manuscript:

ABSV	Average Behavioral Strength Values
ABSVL	Average Behavioral Strength Values in Light Periods
ABSVD	Average Behavioral Strength Values in Dark Periods
AV	Average Velocity
AVL	Average Velocity in Light Periods
AVD	Average Velocity in Dark Periods
D (ABSVL-ABSVD)	Differences between ABSVL and ABSVD
D (AVL-AVD)	Differences between AVL and AVD
